# P07 A qualitative study of patient and clinician views and experiences of rapid respiratory microbiological point-of-care-testing in primary care, nested within a randomized controlled efficacy trial (the RAPID-TEST RCT)

**DOI:** 10.1093/jacamr/dlae217.011

**Published:** 2025-01-23

**Authors:** E Brown, R Clarke, S Abbs, P Mitchell, L Zhu, M Ridd, A Hay, L Yardley

**Affiliations:** Centre for Academic Primary Care, Population Health Sciences, Bristol Medical School, University of Bristol, Bristol, UK; School of Psychological Science, University of Bristol, Bristol, UK; Bristol Trials Centre, Bristol Medical School, University of Bristol, Bristol, UK; Health Economics and Health Policy, Population Health Sciences, Bristol Medical School, University of Bristol, Bristol, UK; Health Economics and Health Policy, Population Health Sciences, Bristol Medical School, University of Bristol, Bristol, UK; Centre for Academic Primary Care, Population Health Sciences, Bristol Medical School, University of Bristol, Bristol, UK; Centre for Academic Primary Care, Population Health Sciences, Bristol Medical School, University of Bristol, Bristol, UK; School of Psychological Science, University of Bristol, Bristol, UK; School of Psychology, University of Southampton, Southampton, UK

## Abstract

**Background:**

Antibiotic prescription is common for respiratory tract infections (RTIs) in primary care, despite evidence to suggest little clinical benefit as many RTIs are viral or self-limiting, with prescribing often attributed to clinical uncertainty regarding microbiological diagnosis. Rapid microbiological point-of-care tests (POCT^RM^) have the potential to improve diagnostic precision by detecting viruses and bacteria from respiratory tract samples and they are proposed as a way to reduce antibiotic prescribing and antimicrobial resistance. However, there is little understanding of patients’ and clinicians’ views of POCT^RM^ use in primary care.

**Objectives:**

To investigate clinicians’ views on how POCT^RM^ could influence clinical decisions and routine practice, and perspectives on how POCT^RM^ may impact the clinician-patient relationship. To explore patients’ and parents’ experiences using POCT^RM^ for RTIs and their views on how POCT^RM^ may influence treatment decisions, symptom management and future consulting.

**Methods:**

Individual, semi-structured interviews were conducted with participants from a multicentre, individually randomized controlled efficacy trial evaluating a multiplex POCT^RM^ for suspected respiratory tract infections in primary care. A total of 18 clinicians (9 GPs/5 Advanced Nurse Practitioners/1 Clinical Pharmacist/2 Paramedics/1 Emergency Care Practitioner) and 30 patients (21 adults/9 parents) were purposively sampled. Interviews were audio-recorded, transcribed verbatim and analysed thematically informed by a realist approach.

**Results:**

Clinicians found POCT^RM^ helpful in guiding prescribing decisions when experiencing diagnostic uncertainty and fear of adverse patient outcomes. Results from POCT^RM^ could help reinforce treatment decisions, support communication with patients and reduce patient anxiety. Nevertheless, clinicians emphasized that clinical judgement remained essential, partly because this POCT^RM^ does not detect typical bacteria. The perceived value, and use of, POCT^RM^ was tied to clinicians’ confidence in making prescribing decisions and managing patient expectations and perceptions about their clinical roles. Clinicians shared concerns that POCT^RM^ could increase future patient consulting and create challenges on practice resources (i.e. cost, time, storage). Patients believed POCT^RM^ results could optimize treatment decisions but emphasized POCT^RM^ should be used alongside clinical judgement. For some patients, additional information from POCT^RM^ reduced the perception that antibiotics were necessary and lowered patient anxiety. Others felt invalidated by POCT^RM^ results not reflecting their illness experience and believed further support alongside POCT^RM^ results would be helpful to understand when antibiotics and consulting were necessary. While POCT^RM^ may reduce re-consulting for the same illness, participants indicated consulting behaviours would persist in the future for self-limiting symptoms or health anxiety. Some participants, who perceived POCT^RM^ to benefit GP practices financially by triaging patients, expressed motivation to consult earlier when symptomatic.

**Conclusions:**

Whilst POCT^RM^ were viewed positively by many patients and clinicians, further research is required to understand their optimal role in clinical decision-making in primary care, and how to reduce the risk of potential adverse outcomes, such as changes to consulting behaviour and an increased burden on practice resources. Complementary strategies alongside POCT^RM^, such as clinician communication skills training and patient education, and clear guidance on implementation, should be explored to enhance POCT^RM^ feasibility and positive outcomes across different primary care settings.

